# Extragenital endometriosis associated with uterine scar defects

**DOI:** 10.1016/j.eurox.2025.100386

**Published:** 2025-03-31

**Authors:** Tilman Born, Katrin Krejci, Maximilian Rauh, Georgia Cole, Maurice Kappelmeyer, Mehmet Vural, Angela Köninger

**Affiliations:** Department of Gynecology and Obstetrics, University of Regensburg, Clinic St. Hedwig, Steinmetzstrasse 1-3, Regensburg 93049, Germany

**Keywords:** Cesarean Scar Defect (CSD), Endometriosis, Uterine Scar Defect, Retrograde Menstruation, Laparoscopy, Cesarean Section Complications, Uterine Niche, Hypermenorrhea, Dyspareunia, Retroflexio Uteri

## Abstract

**Introduction:**

Uterine scar defects result from poor healing of the hysterotomy after cesarean sections (CS), in multiple cases leading to retroflexio uteri and retrograde menstruation. Endometriosis is the probable consequence. Patients often experience overlapping symptoms such as dysmenorrhea, dyspareunia, and infertility.

**Material and methods:**

This study analysed cases of sonographically detected uterine scar defects, subsequently undergoing laparoscopy at the University Clinic St. Hedwig, in Regensburg, between 2020 and 2024. Herefore, surgery reports were reviewed, focusing on extragenital endometriosis, symptoms of Cesarean Scar Disorder (CSD), niche morphology, uterine position, and endometriosis localisation using the #Enzian classification.

**Results:**

Extragenital endometriosis was histologically confirmed in 45 of 94 patients (47.9 %) with symptomatic or large uterine niches. A significant association was found between endometriosis and heavy menstrual bleeding (HMB) (p < 0.001) as well as retroflexio uteri (p = 0.036). Symptoms related to CSD did not differ in patients with or without Endometriosis. Endometriotic implants were primarily located in the peritoneum behind the uterus and sacrouterine ligaments, supporting the hypothesis of retrograde menstruation.

**Conclusion:**

There is significant overlap between the symptoms of endometriosis and CSD. Almost half of the patients with a symptomatic niche were found to have endometriosis, whereby the location of endometriosis supports the hypothesis of retrograde menstruation. However, the patient´s history of complaints is not indicative of the diagnosis of endometriosis. Therefore, all patients with CSD should be offered a laparoscopy and endometriosis surgery. All patients with a previous caesarean section presenting with symptoms of endometriosis should be offered standardised and high-quality niche diagnosis and treatment.

## Introduction

Cesarean sections (CS) are among the most common surgical procedures performed globally, and the repercussions and complications that result from it are becoming more widely recognized. One consequence is impaired healing of the uterine incision, leading to the formation of a uterine scar defect, also known as isthmocele or niche, and its associated symptoms, such as postmenstrual spotting and infertility. In a recent modified Delphi study, a group of 31 international experts on niche-related issues outlined the symptoms associated with this condition and termed it cesarean scar disorder (CSD). CSD is defined as a condition that affects premenopausal women with a niche combined with several clinical symptoms that occurred first or worsened after a CS [1]. Jordans et al. define a niche as a depression present at the location of the caesarean section scar, with a minimum depth of 2 mm measured in a uterine cavity containing fluid or using contrast medium [2]. In several cases, a significant anterior wall defect causes a deformity as severe as a retroflexio uteri. Retrograde menstruation is common in these cases [3]. In 1927, Sampson proposed that the initial stage in the development of endometriosis involves the retrograde flow of menstrual blood containing full endometrial tissue through the fallopian tubes into the peritoneal cavity [4].

Niches can present with a variety of related symptoms, which emphasizes how important precise diagnosis is to adequately address patients’ concerns. Common symptoms include bleeding irregularities, particularly postmenstrual spotting, dysmenorrhoea, dyspareunia, chronic lower abdominal or pelvic pain, secondary infertility, and complications during gynecological procedures such as embryotransfer, caused by the atypical uterine position. Niches also raise the risk of obstetric problems such as placenta accreta spectrum diseases, uterine rupture, and scar pregnancy [5], [6], [7]. A large cesarean scar defect is classified as a residual myometrial thickness (RMT) of < 50 % of the adjacent myometrium detected by sonography. There appears to be correlation between the width of the scar defect and the severity of the symptoms, as it was shown that the width of the scar defect was significantly greater in women with postmenstrual spotting, dysmenorrhoea, and chronic pelvic pain [8], [9], [10], [11].

Endometriosis is a disease characterised by variations in its symptoms and patterns of spread within the peritoneal cavity. Patients typically present with lower abdominal pain along with dysmenorrhea and/or infertility, although some individuals may also be asymptomatic [12], [13], [14]. The #Enzian system has been established in most European countries for the classification of endometriosis [15].

Given the overlapping symptoms, the implantation theory, and the higher probability of retrograde menstruation with larger niches, endometriosis is likely a secondary disease.

The histopathological diagnosis of endometriosis relies on the identification of ectopic endometrial glands and stroma outside the uterine cavity, typically confirmed through biopsy obtained during laparoscopic surgery. Key diagnostic features include the simultaneous presence of both endometrial glands and stroma in the ectopic tissue. Supporting findings such as chronic inflammation and the presence of hemosiderin-laden macrophages are commonly observed due to repeated hemorrhage in the lesions. Immunohistochemical staining can aid in the diagnosis, with CD10 positivity serving as a reliable marker for endometrial stromal cells. Endometrial glands may also express estrogen and progesterone receptors. These markers are particularly useful in challenging cases, where the identification of endometrial components is difficult due to factors such as hormonal treatment or atrophic changes in the glands [16].

Hence, the aim of this case-contol study is to explore the association between morphology, severity and symptoms of niches and the presence of associated endometriosis implants, as well as their location.

## Material and methods

Women with a uterine scar defect who underwent laparoscopic surgery at the Department of Obstetrics and Gynecology at the University Clinic St. Hedwig in Regensburg, Germany, between 2020 and 2024 were included. In all participants, CSD was present according to the following diagnosis criteria: presence of a niche according to [2] in combination with at least one primary symptom, or at least two secondary symptoms. Primary symptoms were defined as postmenstrual spotting, pain during uterine bleeding, difficulties during embryo transfer and an unexplainable secondary sterility in combination with intrauterine fluid. Secondary symptomes are dyspareunia, abnormal vaginal discharge, chronic pelvic pain, odor associated with abnormal blood loss, secondary unexplained infertility, secondary infertility despite of artificial reproductive techniques (ART), a negative self-immage, avoidance of sexual intercourse and discomfort during participation in leisure activities [1]. Addionally, for exact diagnosis of CSD, symptoms have to occur for the first time or worsen after a CS and last at least three months or more after a caesarean delivery [1]. Methodological procedures of this clinical study were reviewed and approved by the Ethics Committee of the University of Regensburg, Germany (22–2289–101).

All cases were analysed for histologically confirmed extragenital endometriosis. Herefore, surgery reports as well as laparoscopic photographs, medical reports and ultrasound findings were analysed. The following data was collected for each patient: age, parity, gravidity, height, weight, body mass index (BMI), number of cesarean sections, stage of delivery at cesarean section, actual complaints according to the symptoms of Cesarean Scar Disorder [1], morphology of the niche (see below), position of the uterus and location of endometriosis implants according to the #Enzian classification.

**Fig. 1:**
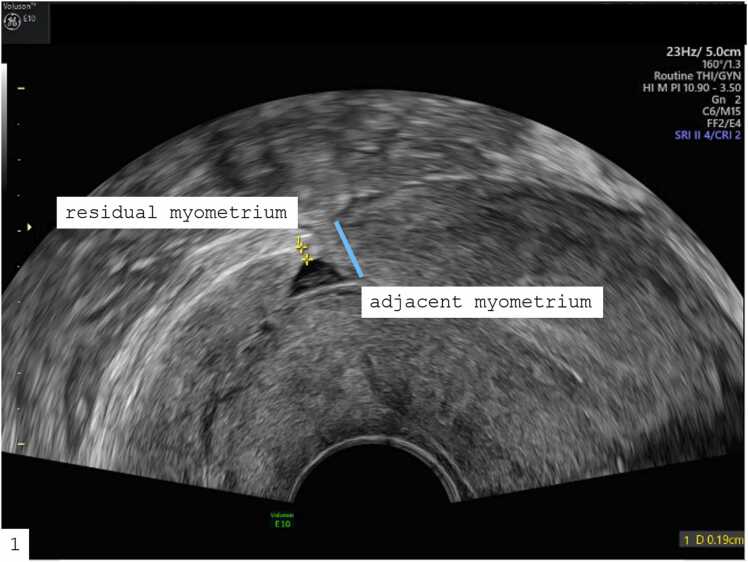
Uterine niche with measurements of the residual and adjacent myometrium.

Transvaginal sonography (TVS) was performed in every patient who presented with suspected CSD. As a rule, the follicular phase of the menstrual cycle was chosen as the timeframe of examination. Patients who were taking hormone therapy or contraceptives were instructed to stop, and were subsequently evaluated no earlier then the second menstrual cycle. For the majority of patients, hysterosonography utilizing the highly echogenic medium ExEm-Foam® was used in order to achieve the highest sensitivity in finding a uterine niche. In patients with a complete dehiscence of the anterior uterine wall containing fluid, native TVS was performed solely. The examination focused on the lower uterine segment adjacent to the uterine cavity, the tubal filling, as well as on all other pelvic organs, such as the ovaries and sonographic signs of endometriosis. All examinations were performed using a high-end ultrasound machine (GE Voluson™ E10). Data documentation was performed using GE Healthcare's ViewPoint 6™ software for data collection. Adhering to the principle of dual assessment, examinations were supervised by a senior consultant with DEGUM II certification (Deutsche Gesellschaft für Ultraschall in der Medizin) in obstetric and gynecological ultrasound. If a niche was identified, it was visualized in all planes following standardised protocols, and its precise size and extension were measured according to Jordans' criteria [2] (Fig. 1). This included assessment of niche depth in mm, RMT and adjacent myometrial thickness (AMT, which is RMT plus niche depth), as well as morphological aspects of the defect, such as included cysts within the scar, were documented.

Only patients without a history of endometriosis before their previous pregnancy were included. Additionally, only patients with symptoms of CSD were included. The inclusion of asymptomatic patients with a niche identified solely during routine gynecological examinations but without symptoms was limited to cases involving a complete anterior uterine wall defect, which carries the highest risk of developing symptoms of CSD in the event of fertility desire or discontinuation of current hormone therapy.

All patients included underwent laparoscopic surgery as part of a scar repair surgery. Patients with symptomatic niches but RMT of > 2 mm and simple niches (explanation?) were treated by hysteroscopy and therefore not included in the study. The standardised surgical procedure in cases of a residual myometrial thickness of less than 2 mm niches or large defects that were not successfully treated by hysteroscopy, consisted of diagnostic hysteroscopy, laparoscopy, dissection of the bladder followed by a minilaparotomy and excision of the scar tissue and closure of the hysterotomy by a multiple single knot closure. In a small cohort, robotic scar repair was performed using the DaVinci® SI system (4/94). The efficacy of the surgical technique will be published and discussed elsewhere; therefore, we do not aim to evaluate the surgical technique, in this study.

Macroscopic lesions suspected of being endometriosis were completely excised or coagulated, and a biopsy was performed to confirm the diagnosis.

During the operation, endometriosis was described according to the #Enzian classification. All findings were photodocumented for pathological review. The diagnosis and treatment of endometriosis was conducted by a highly experienced surgical team with at least one member certified by SEF (Stiftung Endometrioseforschung).

Descriptive statistics for patient demographics and ultrasound parameters were recorded in an Excel file. Statistical analysis was performed using R version 4.4.0 and the gt summary package version 1.7.2. The Wilcoxon rank-sum test was used to compare two independent groups using quantitative data. For the analysis of qualitative factors, either Pearson's Chi-squared test or Fisher's exact test was used, depending on the characteristics of the data. Statistical tests with p-values below 0.05 were considered significant.

Quantitative data was presented as mean, standard deviation (SD), median and interquartile range (IQR). For gravidity and parity, the median and interquartile range were reported. For qualitative factors, both absolute and relative frequencies were reported.

## Results

94 patients were included, of whom 45 (47,9 %) were found to have endometriosis. All patients underwent a laparoscopic scar repair with mini-laparotomy (n = 90) or robotic repair (n = 4). The baseline characteristics of the patients are demonstrated in Table 1. There was no significant difference between the two groups in terms of age, gravidity, parity, height, weight, BMI, number of cesarean sections (CS), elective and secondary CS.

**Table 1:** Baseline characteristics of patients with and without endometriosis.

**Characteristics**	**No endometriosis** N = 49^a^	**Endometriosis** N = 45^a^	**p-value**
Age			> 0.9^b^
Mean (SD)	35.6 (4.7)	35.8 (3.7)	
Median (Q1 - Q3)	36.0 (32.0–39.0)	36.0 (34.0–38.0)	
Gravidity			0.7^b^
Median (Q1 - Q3)	1.00 (1.00–2.00)	1.00 (1.00–2.00)	
Parity			0.058^b^
Median (Q1 - Q3)	2.00 (1.00–2.00)	1.00 (1.00–2.00)	
Height (cm)			0.078^b^
Mean (SD)	164.3 (5.8)	167.0 (6.3)	
Median (Q1 - Q3)	165.0 (160.0–168.0)	167.0 (162.0–172.0)	
Weight (kg)			0.5^b^
Mean (SD)	76 (19)	73 (15)	
Median (Q1 - Q3)	75 (61−86)	73 (62−80)	
BMI (kg/m²)			0.10^b^
Mean (SD)	28.2 (6.5)	26.1 (5.1)	
Median (Q1 - Q3)	26.9 (24.0–30.7)	25.1 (22.6–28.6)	
Number of cesarean sections in the past (CS)	1.00 (1.00–2.00)	1.00 (1.00–1.00)	0.4^b^
Elective CS	13 (28 %)	15 (37 %)	0.4^c^
Secondary CS	15 (33 %)	16 (39 %)	0.5^c^

a Median (Q1 - Q3); n (%)

b Wilcoxon rank sum test

c Pearson's Chi-squared test

The symptoms and aspects of the niche are demonstrated in Table 2. A Pearson’s chi-square test indicated that there is a significant relationship between endometriosis and heavy menstrual bleeding (HMB), X2 (1, N = 93) = 11.5, p < 0.001. Endometriosis patients (17 (38 %)) were more likely to have HMB compared to patients without endometriosis (4 (8.3 %)).

**Table 2:** Symptoms and morphology of the Uterine Niche in Patients with and without Endometriosis.

**Symptoms/morphology of niche**	**No endometriosis** N = 49^a^	**Endometriosis** N = 45^a^	**p-value**
History of recurrent miscarriage^b^	2 (4.1 %)	0 (0 %)	0.5^c^
History of scar pregnancy	2 (4.1 %)	0 (0 %)	0.5^c^
Bleeding disorders	19 (39 %)	24 (53 %)	0.2^d^
Secondary infertility	22 (45 %)	26 (58 %)	0.2^d^
Postmenstrual spotting	21 (43 %)	21 (47 %)	0.7^d^
Premenstrual spotting	2 (4.1 %)	2 (4.4 %)	> 0.9^c^
Pelvic pain	10 (20 %)	7 (16 %)	0.5^d^
Dyspareunia	5 (10 %)	3 (6.7 %)	0.7^c^
Dyschezia	0 (0 %)	1 (2.2 %)	0.5^c^
Dysuria	0 (0 %)	1 (2.3 %)	0.5^c^
Heavy menstrual bleeding	4 (8.3 %)	17 (38 %)	**< 0.001**^d^
Dysmenorrhea	17 (35 %)	20 (45 %)	0.3^d^
Retroflexio uteri	16 (35 %)	25 (57 %)	**0.036**^d^
Presence of cysts within the niche	4 (15 %)	1 (2.9 %)	0.2^c^
Residual myometrium (mm)			0.8^e^
Mean (SD)	1.11 (1.25)	1.30 (1.46)	
Median (Q1 - Q3)	0.80 (0.00–2.00)	1.10 (0.00–2.50)	
Adjacent myometrium (mm)			0.8^e^
Mean (SD)	9.14 (1.89)	9.38 (2.18)	
Median (Q1 - Q3)	9.10 (7.90–10.20)	8.90 (7.60–10.90)	
Niche depth (mm)			> 0.9^e^
Mean (SD)	8.03 (2.24)	8.09 (2.42)	
Median (Q1 - Q3)	7.90 (6.70–9.40)	8.10 (6.30–9.40)	
Proportion niche (mm)			0.8^e^
Mean (SD)	0.87 (0.14)	0.86 (0.15)	
Median (Q1 - Q3)	0.91 (0.79–1.00)	0.88 (0.76–1.00)	

a n (%)

b Defined according to WHO: 3 consecutive miscarriages < 20 weeks of gestation

c Fisher's exact test

d Pearson's Chi-squared test

e Wilcoxon rank sum test

**Fig. 2:**
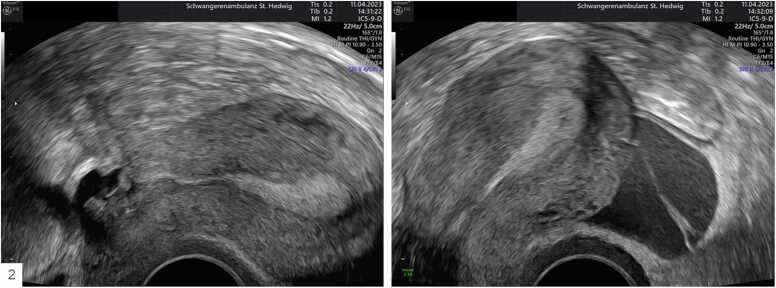
Left: Anterior uterine wall defect resulting in retroflexio uteri Right: Sonographically visible retrograde menstruation.

A significant association between retroflexio uteri and endometriosis was found (X² (1, N = 90) = 4.4, p = 0.036). Endometriosis patients (25 (57 %)) were more likely to have retroflexio uteri compared to patients without endometriosis (16 (35 %)) (Fig. 2). In 4 patients, a severely distorted uterus was observed due to a pronounced defect, which could not be classified as either anteflexio or retroflexio.

**Fig. 3:**
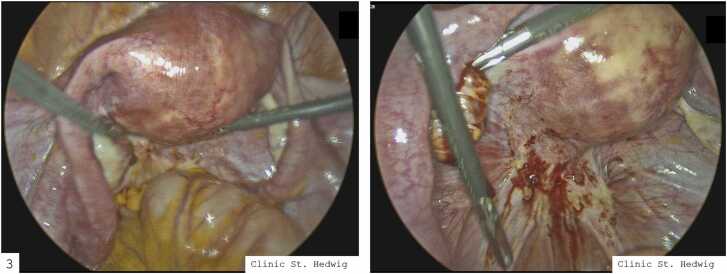
Laparoscopic finding of extragenital endometriosis in a niche patient with retroflexio uteri.

Table 3 gives an overview in terms of the localisation of the endometriosis in combination with the #Enzian classification. Localisation A describes deep infiltrating endometriosis affecting the rectovaginal space, the vagina, and the retrocervical area. Localisation B represents deep infiltrating endometriosis involving the sacrouterine ligaments, cardinal ligaments, and the pelvic sidewall. Localisation C represents deep infiltrating endometriosis of the rectum. P represents the total diameter of peritoneal endometriosis lesions and summarises the presence of peritoneal endometriosis within the pelvis. O represents the total diameter of endometriosis involving the ovaries. Finally, T refers to the involvement of the tubal-ovarian complex, reflecting the condition of both the fallopian tubes and the ovaries in the context of endometriosis (Fig. 3).

**Table 3:** Localisation of endometriosis implants in patients with uterine scar defects.

**Localisation**	**N = 45**^*1*^
Deeply infiltrative	10 (22 %)
Localisation A	3 (6.7 %)
Localisation B	7 (16 %)
Localisation C	1 (2.2 %)
Localisation P	40 (89 %)
Localisation O	10 (22 %)
Localisation T	6 (13 %)
Pouch of Douglas	28 (62 %)
Ligg. sacrouterina	20 (44 %)
Pelvic wall/Pelvic peritoneum	13 (29 %)
Ovarian pouch	11 (24 %)
Endometrioma/ovary	10 (22 %)
Fallopian tube	4 (8.9 %)
Bladder peritoneum	5 (11 %)
Ureter	0 (0 %)
Sigma/Rectum	3 (6.7 %)
^*1*^n (%)

## Discussion

This is the first study evaluating the association between sonographic aspects, severity, and symptoms of a niche and associated endometriosis implant location. We were able to demonstrate that endometriosis could be found in 47.9 % of patients presenting to our clinic between 2020 and 2024 with a symptomatic caesarean scar defect and indication for a laparoscopic repair or asymptomatic complete anterior uterine wall dehiscence.

Patients with uterine scar defects and endometriosis exhibited a higher prevalence of retroflexio uteri (35 % vs. 57 %). This finding is consistent with previous studies, which have demonstrated an increased incidence of retroflexio uteri in individuals with endometriosis [17], [18]. We strongly hypothesize that the uterine wall defect causes the retroflexio due to interruption of the wall structure resulting in weakening of the tissue.

Furthermore, the study showed an association between HMB and endometriosis in patients with uterine scar defects. While most symptoms did not differ significantly between patients with and without endometriosis, HMB was significantly more common in endometriosis patients (8.3 % vs. 38 %). This finding is consistent with other studies identifying HMB or menorrhagia as a characteristic symptom in patients with endometriosis [19], [20].

The most intriguing, yet unexpected, discovery is the almost total overlap between endometriosis and CSD symptoms, including infertility, dyspareunia, dysmenorrhea, and persistent pelvic discomfort. Patients with a niche present with symptoms that are well known solely for endometriosis. However, whether or not patients with a niche have additional endometriosis does not affect their symptom pattern or severity. Therefore, all patients with a niche should be offered a laparoscopic intervention to exclude or treat endometriosis.

Furthermore, patients with a history of CS who present with typical endometriosis symptoms such as dysmenorrhea, dyspareunia and chronic pelvic pain may alternatively suffer from a uterine scar defect.

Due to the overlap in symptoms between both conditions, there is a risk that a uterine scar defect may go undiagnosed if clinicians focus solely on endometriosis. Therefore, it is important to consider the possibility of a scar defect in patients with a history of CS and typical endometriosis symptoms, allowing for more comprehensive diagnosis and treatment [1], [21], [22]. Consequently, this subgroup of patients should receive not only laparoscopic surgery, but also niche diagnosis and treatment according to current standards.

The localisation of endometriosis implants in this study, along with the absence of old, scarred endometriotic lesions, provides valuable insights into the pathogenesis of endometriosis. The most frequent endometriosis implants were found in the Douglas pouch and the sacrouterine ligaments, consistent with the known distribution of endometriosis in the pelvic region. This seems to differ from patients with endometriosis without a scar defect [23], [24]. Audebert et al. showed the most frequent site of endometriotic lesions being the ovary, followed by the utero-sacral ligaments, the ovarian fossa, the pouch of Douglas and the bladder [25]. In niche patients with huge defects, we strongly expect disturbed uterine contraction of the anterior wall during menstruation, resulting in enhanced retrograde menstruation. In niche patients, we mainly observe endometriosis implants in the Douglas pouch, sacrouterine ligaments and the peritoneum behind the uterus. We hypothesise that this almost uniform distribution of endometriosis implants results from retrograde menstruation following the predetermined anatomical path. Retroflexio uteri in CSD patients refelects the weakness of the anterior uterine wall. CSD patients with endometriosis showed to have retroflexio uteri significantly more often than anteflexio uteri. The pathogenesis of endometriosis involves a complex interplay of retrograde menstruation, coelomic metaplasia, immune dysfunction, genetic susceptibility, chronic inflammation, and hormonal sensitivity. These mechanisms, acting together, help to explain the heterogeneity and systemic nature of endometriosis, highlighting the need for a multifaceted approach to its diagnosis and management [4], [10], [26], [27], [28], [29], [30]. However, it is impossible to prove that the endometriosis discovered laparoscopically during niche repair did not exist prior to the C-section, the biological plausibility indicates that endometriosis developed secondary to retrograde menstruation because the patients only complain of symptoms after the C-section.

The methodology used in this study, including the use of contrast medium based transvaginal sonography and laparoscopy for evaluation, reflects current best practice in the diagnosis and evaluation of cesarean scar defects and associated endometriosis. The standardised protocols for imaging and surgical assessment enhance the reliability and validity of the study findings [2], [10].

## Strength and Limitations

This is the first study to explore the association between morphology, severity and symptoms of a niche and associated endometriosis implants and their location. Despite the valuable insights provided by this study, several limitations warrant consideration. The sample size may reduce the generalizability of the results. Future follow-up cohorts may show divergent results as more women with typical symptoms are referred to specialist centers for niche diagnosis and treatment, due to rising awareness. Furthermore, we only included patients with large sonographic defects requiring laparoscopic and not hysteroscopic repair. In patients with CSD but with residual myometrium > 2 mm and without severe clinical symptoms suspicious for endometriosis, we did not routinely perform a laparoscopy. Therefore, we lack knowledge about endometriosis in patients with symptomatic, but small niches who are only treated with hysteroscopy.

## Conclusion

The study highlights the link between cesarean scar defects and endometriosis, where Patients with a niche and simultaneous endometriosis present with identical symptomes as patients without simultaneous endometriosis This suggests that patients with similar symptoms to endometriosis should be considered for laparoscopic surgery. However, clinical symptoms alone aren't enough to exclude or suspect additional endometriosis, necessitating comprehensive diagnostics for optimal treatment strategies. We will continue researching this field to further explore the connection between defects in the uterine wall, retroflexion and endometriosis development. In further studies, we aim to encompass molecular studies on endometriotic lesions in CSD patients compared with endometriotic lesions in patients without previous labor.

## CRediT authorship contribution statement

**Born Tilman:** Writing – original draft, Validation, Project administration, Investigation, Formal analysis, Data curation, Conceptualization. **Krejci Katrin:** Data curation, Conceptualization. **Rauh Maximilian:** Writing – review & editing, Validation. **Cole Georgia:** Writing – review & editing. **Kappelmeyer Maurice:** Validation, Formal analysis. **Vural Mehmet:** Writing – review & editing, Supervision. **Köninger Angela:** Writing – original draft, Validation, Supervision, Project administration, Methodology, Investigation, Formal analysis, Conceptualization.

## Declaration of Competing Interest

The authors declare that they have no known competing financial interests or personal relationships that could have appeared to influence the work reported in this paper.
